# Perception of English phonetic contrasts by Dutch children: How bilingual are early-English learners?

**DOI:** 10.1371/journal.pone.0229902

**Published:** 2020-03-11

**Authors:** Claire Goriot, James M. McQueen, Sharon Unsworth, Roeland van Hout, Mirjam Broersma

**Affiliations:** 1 Centre for Language Studies, Radboud University Nijmegen, Nijmegen, The Netherlands; 2 Donders Institute for Brain, Cognition, and Behaviour, Centre for Cognition, Radboud University Nijmegen, Nijmegen, The Netherlands; 3 Max Planck Institute for Psycholinguistics, Nijmegen, The Netherlands; Universita degli Studi di Milano-Bicocca, ITALY

## Abstract

The aim of this study was to investigate whether early-English education benefits the perception of English phonetic contrasts that are known to be perceptually confusable for Dutch native speakers, comparing Dutch pupils who were enrolled in an early-English programme at school from the age of four with pupils in a mainstream programme with English instruction from the age of 11, and English-Dutch early bilingual children. Children were 4-5-year-olds (start of primary school), 8-9-year-olds, or 11-12-year-olds (end of primary school). Children were tested on four contrasts that varied in difficulty: /b/-/s/ (easy), /k/-/ɡ/ (intermediate), /f/-/θ/ (difficult), /ɛ/-/æ/ (very difficult). Bilingual children outperformed the two other groups on all contrasts except /b/-/s/. Early-English pupils did not outperform mainstream pupils on any of the contrasts. This shows that early-English education as it is currently implemented is not beneficial for pupils’ perception of non-native contrasts.

## Introduction

When it comes to second language (L2) sound acquisition, it is often claimed that ‘earlier is better’ (see [1] for a review): becoming able to distinguish certain speech sounds that occur in a non-native but not the native language can be very difficult at a later age. In the Netherlands, for example, the premise of ‘the earlier the better’ was why the Dutch Education Council advised the Ministry of Education to lower the starting age of foreign-language education, from the age of ten to—preferably—the age of four [2]. The Council reasoned that older children would be hindered by their first language (L1) when learning a new language, while younger children would not have fully developed their L1 yet and hence could learn a new language more easily and with greater success than older children [2]. Indeed, just like in many other countries in Europe [3], a growing number of Dutch primary schools now provide early-English education, often from the moment children enter primary school [4].

The question that is addressed in the current study is whether this early foreign-language education has a positive influence on children’s L2 speech sound perception. We investigate whether children who receive this kind of education are indeed better able than mainstream pupils to discriminate English phonetic contrasts that are known to be difficult to acquire for Dutch L1 speakers. In addition, we examine whether they can do so as well as bilingual children, that is children who are growing up with both Dutch and English as their native languages.

### Foreign speech perception

Initially, infants can perceive phonetic contrasts of all languages, but within the first year of life that ability diminishes while their perception becomes attuned to the L1. Recent evidence shows that infants already have robust knowledge about the sounds of their L1 by the age of three months [5]. Around the age of six months old, infants become more sensitive to contrasts in their native language(s), and lose their ability to perceive some of the vowel contrasts of foreign languages [6]. For consonants, this happens around the age of eleven months [6,7]. Infants seemingly effortlessly learn the phonetic contrasts of the language they hear [6], and if they receive input in two languages, they will learn the contrasts of both languages [8–12]. This does, however, not mean that infants immediately categorize all sounds of their native language(s) correctly. Research with monolingual English children has shown that five-year-olds are more likely than nine-year-olds to categorize a foreign vowel as a native one, and that both groups are less consistent than adults in categorizing native and non-native vowels [13]. As they grow older, monolingual children become increasingly consistent in phonemic categorization, but even at the age of 12 their performance is not adult-like [14].

It is much more difficult to learn to perceive the speech contrasts of a foreign language at a later age (for reviews see [15–17]). For example, previous research has shown that, notoriously, adult Japanese learners of English have difficulty distinguishing between /r/ and /l/ [18–20], and Dutch learners of English, despite high proficiency in English as an L2, are not as good as native English speakers in perceiving the difference between /ɛ/ and /æ/ [21].

Children are better able than adults to learn foreign speech sounds [19,22–24]. For example, Tsukada et al. [22] compared English speech perception abilities of Korean adults and children between 9 and 17 years old, who had lived in America for either three or five years. After three years of residence, the children’s discrimination abilities were already significantly better than the adults’. Adults’ ability to discriminate between English contrasts did not differ between the groups that had been in America for a shorter or longer time. Children who had resided in America for five years were significantly better able to discriminate English speech contrasts than those who been in America for three years, however, resulting in an even greater difference between the adult and child group when comparing the groups who had resided in America for five years.

As the children in the previous study had extensive exposure to the new language, the question remains whether children are also able to learn phonetic contrasts of a foreign language that they learn in a more formal setting, such as in school. McCarthy, Mahon, Rosen, and Evans [25] investigated four-year-old Sylheti-English sequential bilingual children’s perception of the English voicing contrast in plosives when they were in kindergarten and again one year later. Before entering kindergarten, children were exposed to English less than 20% of the time. In kindergarten the sequential bilingual children were not as proficient as their English monolingual peers in differentiating between voiced and voiceless plosives. After one year, however, their performance had significantly improved and matched that of the monolingual group. In contrast, a study with 11-year-old Turkish-German sequential bilinguals who had been immersed in a German environment from before the age of four showed that their perception of German vowels was significantly poorer than that of German monolingual children [26]. Unlike the bilingual children in the McCarthy et al. [25] study, who were educated in a monolingual programme, the Turkish-German children were educated in a German-Turkish bilingual programme. They thus received less input in their L2 than the Sylheti-English children, but were exposed to it from an earlier age.

In both studies [25,26], children were immersed in their L2 at school, either 50 or 100% of the time. Such extensive L2 exposure is much more than the amount of exposure offered in foreign language programmes at school, such as the ones gaining in popularity in most countries in continental Europe, including the Netherlands [3]. Nowadays, almost one in five Dutch schools start their English lessons from the moment children enter primary school at the age of four. The legally obligatory onset of English education, however, is not until the penultimate year, when children are approximately ten years old [4]. As children in the Netherlands receive on average 60 minutes of English lessons per week, early-English pupils leave primary school after 320 hours of English education, as opposed to 60 hours (45 minutes per week) for pupils who start in the penultimate grade (at age ten) [27]. The idea behind early-English education is that it will benefit pupils’ English proficiency more than mainstream English lessons, but not at the expense of Dutch. Previous research in the Netherlands has indeed shown beneficial effects of early-English programmes for pupils’ English vocabulary [28–30], grammar [28], reading and writing abilities [31], and word pronunciation [32]. Research on Dutch early-English pupils’ abilities to perceive English speech contrasts, however, is not yet available.

International research on early-English pupils’ perception of English contrasts is scarce, too. Jost et al. [33] investigated Swiss-German children’s responses to the English contrast /t/-/θ/: once before starting English lessons, and once a year later, after having received one and a half hours of English lessons per week. Children showed a significant increase in accuracy in discriminating between /t/ and /θ/ after one year of English instruction.

With respect to the Dutch situation, pupils necessarily encounter unfamiliar sound contrasts that they have to learn to distinguish. Nevertheless, during English lessons not much attention seems to be given to the perception of these unfamiliar speech sounds. The goals for English education focus on understanding spoken and written English texts, and being able (and confident) to communicate in English [34]. Consequently, the goal of English lessons in Dutch education is mainly oral proficiency, and especially speaking, listening, and vocabulary development [35]. Activities for young pupils are aimed at learning English in a playful way, for example by the means of songs and stories. For older pupils, from grade five onwards (8-9-year-olds), content learning and language learning are integrated and English is used as the language of communication during other subject lessons. In practice, teachers speak English during those lessons, but pupils hardly speak any English to each other or the teacher [36]. According to the organisation for internationalisation in education, in order to teach in English, teachers’ proficiency in English should be at least at the intermediate level (B2 on the Common European Reference Framework) [37]. In practice, teachers vary in their proficiency levels, ranging from low proficient to (near-)native proficiency [27]. Since children are usually educated by more than one teacher, and a different teacher every school year, the quality of the input in English they receive at school likely varies to a large extent.

The idea behind early-English education is that providing children with lessons in English will ameliorate their proficiency in English. The assumption is that exposing young children to English will foster their language development. As such, children first learn to understand English, and then to speak it themselves. It is only in the higher grades of primary school that they learn to read and write in English [37]. Given that children start with understanding English, and that it seems that especially foreign speech sounds should be learnt early in life in order to learn to distinguish between them [22,23,25], the question is whether the learning activities that pupils in early-English education take part in contribute to the advancement of their perception of L2 speech contrasts. In this study, we investigate whether Dutch children who are exposed to English from a young age in an educational setting are better able to distinguish such English speech contrasts that do not exist in their native language than children in a mainstream program, or not.

### Contrasts of interest

Dutch and English phoneme categories differ in various ways. According to the Perceptual Assimilation Model (PAM), the degree to which L2 learners should be able to learn to perceive L2 contrasts depends on the way in which they differ from the L1 phonetic system [38–40]. The focus of this study is on four English phonetic contrasts that are expected to vary in difficulty for native speakers of Dutch, ranging from very easy to very difficult.

In the first contrast of interest, /b/-/s/, the phonemes differ from each other in place, voicing and manner of articulation. Both English and Dutch have a /b/ and /s/ that are pronounced rather similarly in both languages. (Differences include the VOT of /b/, which is negative in Dutch and around 0 for English). In PAM, such a contrast is called a ‘two category’ assimilation contrast [40]. According to the predictions of PAM, when two non-native phonemes are very similar to two phonemes in the native language, listeners will perceptually assimilate these non-native phonemes to the corresponding native categories. Consequently, pupils should be able to easily discriminate between members of such a contrast. The /b/-/s/ pair was thus included in this study as an easy contrast. At the same time, it served as a control contrast, to ensure that children understood the task.

Second, the /k/-/ɡ/ contrast was expected to be of intermediate difficulty. Dutch as well as English has /k/ and both are pronounced rather similarly (but in English with a long positive VOT, and in Dutch with a 0 to short positive VOT). Contrary to English, Dutch does not have a /ɡ/. According to PAM, the English /k/-/ɡ/ contrast would be a ‘category goodness’ contrast, where two different L2 phonemes are both mapped onto the same L1 category but one is considered to be a better exemplar of that category than the other. According to PAM, recognition of an L2 phoneme should be more difficult when two phonemes are mapped onto the same L1 category than when both phonemes are mapped onto two different L1 categories [38–40]. Previous research has shown that, indeed, native Dutch adults find it difficult to identify the English /ɡ/ [41]. Further, although most Dutch native adults are able to produce word-initial /ɡ/, substitution with [k] or [x] also occurs [42,43]. There are, however, three reasons to expect that the English /k/-/ɡ/ contrast might be easier for Dutch listeners to differentiate than other category goodness contrasts. First, in Dutch, whereas there is no velar voicing contrast (/k/-/ɡ/), bilabial (/p/-/b/) and alveolar plosives (/t/-/d/) are contrasted in voicing. In other words, /ɡ/ is missing from a consonant system which does contain /b/ and /d/, and hence there is a *system gap* [44]. Second, the /ɡ/ does occur in Dutch in loan words [42,43,45] and, third, it occurs as an allophone of /k/. PAM does not explicitly discuss the possible effect of such L1 experience with (features of) sounds that are not present as a phoneme in the L1, and its predictions for category goodness contrasts seem to assume that the L2 learners are completely inexperienced with respect to one of the members of the contrast. This does not hold, however, for the /ɡ/ in Dutch. The first reason is that Dutch native listeners *do* have experience with the voicing contrast, in other places of articulation. Second, they also have some experience with /ɡ/ specifically, since it occurs in loanwords and as an allophone of /k/. It is therefore expected that Dutch listeners might find it easier to perceive the English /k/-/ɡ/ contrast than a category goodness contrast that they do not have such L1 experience with. Indeed, it has been shown that Dutch listeners tend to identify /ɡ/ more accurately than consonants with features that do not occur at all in Dutch, like /θ/ or /ð/ [41].

The third contrast we included was therefore /f/-/θ/. Just like /k/-/ɡ/, /f/-/θ/ can be considered to be a ‘category goodness’ contrast, as Dutch has an /f/ fairly similar to the English one, but no /θ/. Previous research has shown that Dutch adults have difficulty identifying the English consonant /θ/. They often perceive /θ/ to be similar to /t/, /s/, or /f/ [41,46]. The predictions of PAM for /k/-/ɡ/ and /f/-/θ/ are similar. We expected the /f/-/θ/ contrast, however, to be more difficult for Dutch learners of English than the /k/-/ɡ/ contrast. First, the lack of /θ/ in Dutch is not a system gap, as Dutch has no dental place of articulation in the consonant system. Furthermore, unlike /ɡ/, /θ/ does not occur in the pronunciation of loanwords or as an allophone. Further the perception of /θ/ is intrinsically difficult; even native speakers of American English found /θ/ to be confusable with /f/ [41,47].

The final contrast we included was /ɛ/-/æ/. Whereas the English vowel inventory includes both open midfront unrounded vowels, Dutch only has /ɛ/. The Dutch /ɛ/ lies between the English /ɛ/ and /æ/, and is typically lower than the English /ɛ/. Following PAM predictions for what is called a ‘single category’ assimilation contrast, Dutch native speakers should find it very difficult to distinguish between/ɛ/ and /æ/, as shown previously indeed [21]. According to PAM, both /ɛ/ and /æ/ will be perceived as an exemplar of the Dutch /ɛ/. Given that unlike the /k/-/ɡ/ or /f/-/θ/ contrast, neither member will be a better exemplar of the Dutch category, Dutch learners of English are expected to have the most difficulty distinguishing between both members of this contrast.

Whereas Dutch children learning English may have difficulty distinguishing between the English contrasts mentioned above, that does not imply that they will not be able to learn to distinguish those contrasts at all. For the category goodness contrast, when two L2 phonemes map onto the same L1 category while only one L2 phoneme is a good exemplar of that L1 category, learners may eventually form a new category for the L2 phoneme that does not exist in their native language, and learn to discriminate between the two members of the L2 contrast [40]. It is therefore expected that Dutch children may initially not perceive the difference between /k/ and /ɡ/, or /f/ and /θ/, but that they will learn to distinguish these phonemes after several years of learning English. Our expectation is that Dutch children are able to distinguish between /k/ and /ɡ/ earlier than between /f/ and /θ/, given that they encounter voicing distinctions in the Dutch language too, as well as instances of /ɡ/ in loanwords and as allophones of /k/.

For the single category assimilation contrast, when two L2 phonemes are mapped onto the same L1 category but neither of them is a good exemplar of that L1 category, learners may not overcome their difficulties in discriminating between the two L2 sounds [40]. It is therefore expected that Dutch L2 learners’ difficulty to distinguish between /ɛ/ and /æ/ is not limited to the initial stages of L2 acquisition, but may persist even after several years of L2 learning [21,41].

### This study

One of the ideas behind early-English education is that such a programme will benefit pupils’ English language skills, while at the same time their Dutch language skills develop just like those of their monolingually educated peers. The main research question of this study was whether early-English pupils are better able to discriminate between English phonemes than pupils from mainstream schools, and whether early-English pupils’ performance is comparable to that of children growing up with both English and Dutch as their home languages.

The participants differed in their experience with English. The bilingual children grew up with both English and Dutch as their native languages. The early-English and mainstream pupils were raised in Dutch. Both are expected to be exposed to English via media [48]. For example, English movies are not dubbed but rather subtitled. For early-English pupils, English lessons started at the start of primary school (i.e., at the age of four). For mainstream pupils, English lessons started near the end of primary school (at the age of ten). Since English lessons generally do not last more than one hour per week, even with out-of-school exposure to English, pupils had much more limited exposure to English than the children that were being raised bilingually. This study examines whether limited L2 exposure in a non-naturalistic setting is related to improved perception of L2 speech contrasts, and if so, how long it takes for such an improvement to become detectable. By investigating to what extent children are able to learn to discriminate non-native speech sounds under such conditions, this study will thus contribute to knowledge about the plasticity of the speech perception system.

We investigated speech perception abilities in three age groups: children who had just started primary school (4–5 year olds), children who were halfway through primary school (8–9 year olds), and children who were at the end of primary school (11–12 year olds). As is often the case, age at testing and amount of L2 exposure are intertwined [49]; we do not attempt to separate the two from each other nor do we want to make any claims about whether starting at an earlier age is better for phonological learning. Age of onset is the same for all the early-English children participating in this study (i.e., 4 years). The goal of including pupils from different age (at testing) groups was to investigate the effect of limited English instruction after several years. Since early-English pupils generally have one hour of English lessons per week, the 4-5-year-old mainstream and early-English pupils were expected to be comparable in amount of instruction (i.e., none to very limited amount of instruction). After five years, 8-9-year-old early-English pupils would have had an estimated 200 hours of English instruction as opposed to no instruction for their peers in mainstream schools. By the end of primary school, 11-12-year-old early-English pupils would have had an estimated 320 hours of English education, as opposed to 60 hours for mainstream pupils.

Three research questions were investigated. The first one was whether early-English pupils outperform mainstream pupils on the perception of the non-native contrasts, and whether bilingual children outperform early-English pupils. We hypothesized that the bilingual children should outperform the two other groups, and if anything, the early-English group should outperform the mainstream pupils.

Second, we asked whether older pupils have better speech perception abilities than younger pupils. We hypothesized that on all contrasts and for all three groups of children, older children would perform better than younger children. This was expected because older children generally show better phoneme categorization than younger children [13,14], and moreover the older groups in this study have had more exposure than the younger groups.

Third, we examined whether the four different speech contrasts investigated here differed in difficulty level for native speakers of Dutch. The hypothesis was that the degree of similarity between the English and Dutch phoneme inventory should predict the perceptual difficulty, especially for the mainstream and early-English children. Following the predictions based on Best and Tyler [40] discussed earlier, we expected pupils to find the /b/-/s/ contrast relatively easy, the /k/-/ɡ/ contrast more difficult, the /f/-/θ/ contrast even more difficult, and the /ɛ/-/æ/ contrast the most difficult.

## Materials and methods

This research was approved by the Ethics Assessment Committee Humanities of Centre for Language Studies at Radboud University, Nijmegen. The case number is 8420. Active written consent was taken from participants and data were analyzed anonymously.

### Participants

Three groups of children participated: a control group (*n* = 48), a group of early-English pupils (*n* = 64), and a group of bilingual children (*n* = 48; see also Table 1). An additional 19 children had been tested, but their data were removed because of a technical error of the speech perception task (*n* = 1), an odd response pattern (constantly pressing the same button, or constantly alternating two buttons) on the speech perception task (*n* = 9), or because they did not seem to understand the speech perception task (*n* = 9; see also the Results section for a more detailed procedure of data removal).

**Table 1:** Percentage correct on each of the contrasts, for each type of English acquisition separately.

			4-5-year-olds			8-9-year-olds			11-12-year-olds		
			Mainstream	Early-English	Bilingual	Mainstream	Early-English	Bilingual	Mainstream	Early-English	Bilingual
Contrast (prop. correct)		*N*	15	21	12	15	19	18	18	24	18
	/b/-/s/	*M*	.81	.84	.89	.96	.98	.96	.99	.98	1.0
		*(SD)*	(.13)	(.13)	(.11)	(.06)	(.04)	(.06)	(.03)	(.04)	(.00)
	/k/-/ɡ/	*M*	.54	.60	.68	.71	.78	.90	.80	.84	.94
		*(SD)*	(.14)	(.18)	(.09)	(.12)	(.15)	(.13)	(.13)	(.11)	(.07)
	/θ/-/f/	*M*	.47	.56	.60	.63	.55	.66	.59	.61	.69
		*(SD)*	(.10)	(.13)	(.12)	(.14)	(.10)	(.14)	(.18)	(.16)	(.17)
	/ɛ/-/æ/	*M*	.57	.53	.68	.52	.55	.71	.51	.47	.81
		*(SD)*	(.12)	(.12)	(.12)	(.10)	(.05)	(.20)	(.09)	(.08)	(.17)

The Dutch primary school system consists of eight grades. The control group included children attending a mainstream Dutch school in which English lessons started in grade six or seven (when pupils are nine or ten years old; two schools). Early-English pupils attended a school in which English lessons started in the first grade (kindergarten, i.e., when pupils are four years old; three schools). All early-English schools had a certificate from an independent organization that they taught English for at least 60 minutes per week, and that teachers had at least a B2- (high intermediate) level of English (except for writing, for which B1 [low intermediate level] had been deemed sufficient by the certification organization). The schools had been early-English schools for at least eight years, such that the children who were now in the final grade had started their English education when they entered primary school. Schools were recruited via telephone. If they were interested in participating, they received a document with more information about the study. Schools voluntarily agreed to participate.

Bilinguals were children who had at least one parent who was a native speaker of English. All bilingual children had started to learn Dutch before the age of four, for example because they went to Dutch day care. Three bilingual children were exposed to one additional language (beyond Dutch and English), and one child to two additional languages.

Children had no known hearing or developmental disorders. All parents gave informed consent for participation, and were also asked to fill in a questionnaire about out-of-school exposure to English.

### Instruments

#### XAB non-word discrimination task

Participants were presented with an XAB task in which three non-word stimuli were presented. Children had to indicate which of these stimuli (the second or third) matched the first stimulus (X). The XAB task was presented as a game [50], programmed with Presentation software (version 14.7) from Neurobehavioral Systems. The task consisted of 64 trials, which were administered in two sessions. The first session was preceded by an explanation and six practice trials. The second session was preceded by four practice trials. In the practice trials, a cartoon of one large and two smaller dinosaurs showed the large dinosaur saying a bisyllabic non-word, not containing the contrasts of interest, and subsequently, the two smaller dinosaurs each saying a non-word, one of which was the same as that spoken by the larger dinosaur, and one different. The child was instructed to decide which smaller dinosaur correctly repeated the larger dinosaur and to indicate their response by pressing a key on the laptop. The button ‘A’ corresponded to the left animal, and the button ‘L’ to the right one. Buttons were marked with stickers, to remind children of the response buttons. Children received feedback on their performance. If they pressed the wrong button, the dinosaur that corresponded to the correct button started crying. The large dinosaur would say that the child pressed the wrong button and encouraged the child to press the correct button. If children pressed the correct button, the corresponding dinosaur would jump up and down while throwing around confetti. The large dinosaur would also verbally confirm that the correct button was pressed.

**Fig 1:**
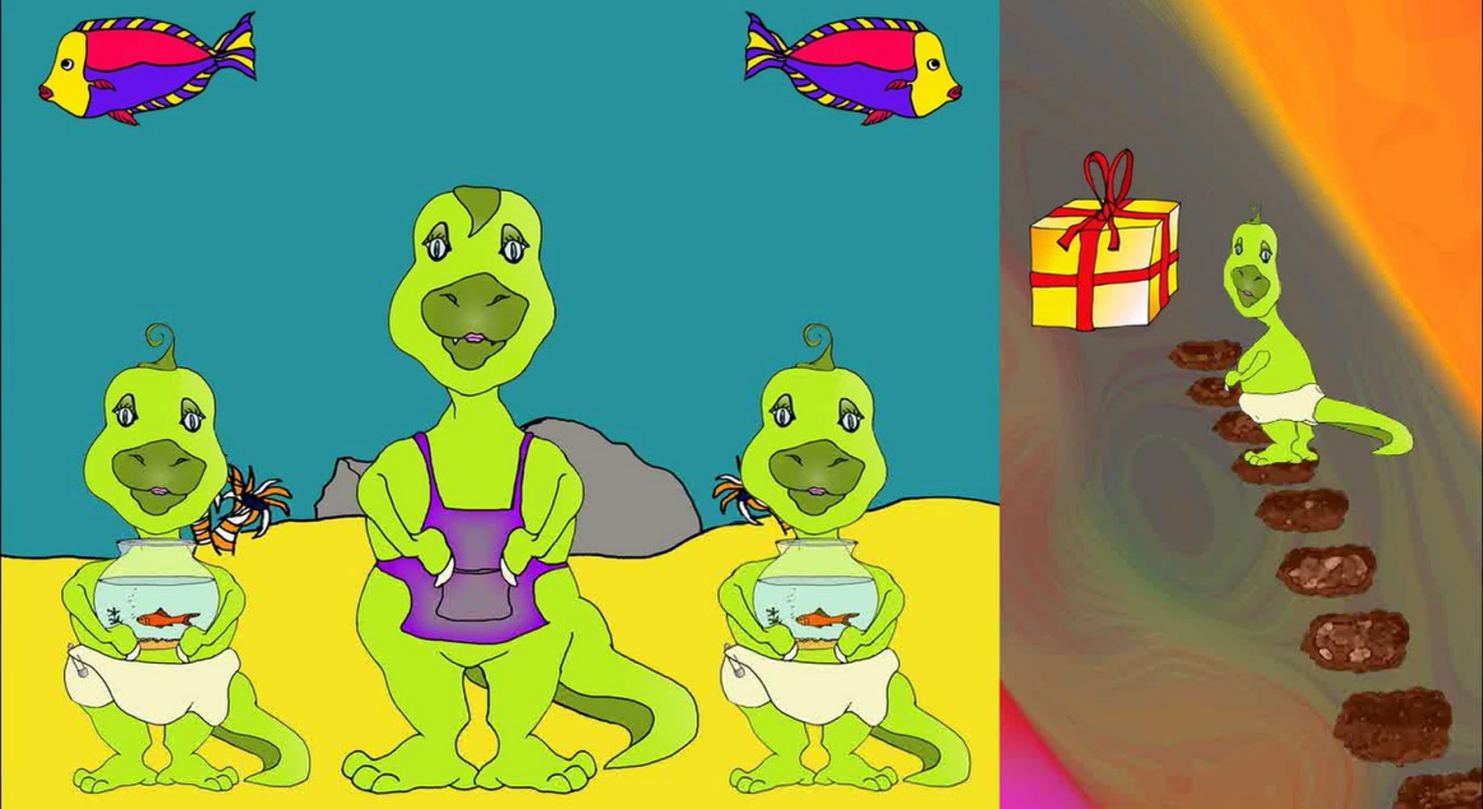
Screenshot of an experimental trial in the XAB task.

In the experimental trials in which the four contrasts were tested, the cartoon of the three animals covered two-thirds of the screen, and a cartoon of a small animal on a staircase covered the other one-third (see Fig 1). For the first two blocks of each session the animals were dinosaurs, and for the last two blocks of each session they were pandas. In the experimental trials, children no longer received feedback. As an incentive, once children pressed a button, the animal on the staircase would jump up one step, no matter if the child performed correctly. In both the practice and the experimental trials, it was always the left animal who first imitated the larger animal. Children could press a button after the second small animal had spoken.

#### Stimuli

The stimuli consisted of eight minimal pairs of non-words. Every minimal pair was presented eight times during the experiment. The stimuli were disyllabic non-words that were phonotactically legal in English (and Dutch). The consonant pairs /b/-/s/, /k/-/ɡ/ and /f/-/θ/ were presented in two different VCV carrier sequences (/әCi/ and /әCa/). The vowel contrast /ɛ/-/æ/ was presented in two different VCVC carrier sequences (/әpVp/ and /әtVt/).

Recordings of the stimuli were made by three female native speakers of Standard American English, each to represent the voice of one designated animal. For each speaker, each stimulus was recorded four times while they read the stimuli one by one in random order, in a clear citation style. Recordings were made in a soundproof booth. The sampling rate at recording was 44.1 kHz.

The 64 trials were presented in eight blocks of eight items each. Each contrast appeared twice in each block. Trials were presented in pseudo-random order: No more than one trial targeting the same contrast and no more than two trials with the same carriers followed each other, and the same animal did not say the correct answer more than three times in a row. The number of times each of the smaller animals correctly repeated the larger animal was counterbalanced across the two smaller animals within blocks.

#### Vocabulary

To investigate to what extent children had knowledge of both languages, and to examine to what extent the three type-of-English-acquisition groups differed from each other in their knowledge of these languages, English and Dutch vocabulary were measured. English vocabulary was measured with the PPVT-4 [51] and Dutch vocabulary with the PPVT-III-Dutch [52]. The test-retest reliability coefficients for children aged between 4;0 and 13;0 years are very high, ranging from .91 to .94 for the English version [51], and between .91 and .96 for the Dutch version [52]. In both tests, the child is presented with a spoken word and is asked to indicate the corresponding picture out of a set of four. The English test consists of 228 items, the Dutch one of 204. Items are grouped in sets of 12. The rules for administration as given in the manual were followed. Testing stopped if children made more than the maximum number of errors in one set (eight for the English and nine for the Dutch PPVT). The score was computed as the number of correctly performed items.

#### Intelligence

To control for possible differences in intelligence, the ‘Matrix Reasoning’ subtest of the Wechsler Nonverbal Scale of Ability was administered [53]. The subtest consists of 41 matrices of which one piece is missing. The child is asked to indicate the correct piece amongst four or five alternatives. Testing is stopped when four or five errors are made in five consecutive items. The total number of correct items determines the score.

### Procedure

Children were tested individually in a quiet room, either at school (early-English and mainstream pupils) or at home (bilingual children). For the participants tested at school, the tasks were presented in two sessions of 25 minutes each. In the first session, the PPVT-4 was administered first, followed by the first part of the XAB task (4 blocks, 32 trials). Session two started with the second part of the XAB task, followed by Matrix Reasoning and then the PPVT-III-NL. The bilingual children took part in a larger study in which multiple tasks were administered which are not reported here. For them, like for the early-English and mainstream children, the XAB task was always administered at the end of the first session and at the beginning of the second session, the PPVT-4 in the first session, and the PPVT-III-NL in the final session. For all (mainstream, early-English, and bilingual) children except four, the two sessions were administered on two separate days, which were on average 8 days apart (*SD* = 11; of those four exceptional children, three did both sessions on the same day, and one participated only in the first session). For five bilingual children, the matrix reasoning task was not (or not correctly) administered, for three bilingual children, the PPVT-4, and for two bilingual children the PPVT-III-NL was not (correctly) administered. Scores for these children on these tasks are missing. Responses to all tasks were registered on a laptop (XAB task) and/or noted down by the experimenter (PPVTs and Matrix Reasoning).

## Results

### Data screening XAB task and differences in background variables

Each individual participant’s response pattern was investigated. We removed data where children either consistently pressed the left or the right button, or pressed the two buttons in strict alternation. First, we removed all the data of children who showed such a pattern throughout the entire task (*n* = 9; 2 early bilinguals, 4 early-English, and 3 mainstream pupils; all were 4-5-year-olds). Next, we removed data of individual sessions that showed such a pattern: The first session was removed for one participant from a mainstream school, and the second session for three early-English and two mainstream-school pupils, all in the 4–5 year old group. After that, we removed data of individual blocks with such a response pattern. In total, 23 blocks were removed, from seven early-English and nine mainstream-school pupils, one in the 11–12 year old group, two in the 8–9 year old group, and 13 in the 4–5 year old group. Next, we removed trials on which participants had RTs that were more than 2.5 *SD*s above their own mean RT. Due to a technical error in the task, the reaction times of one participant (4–5 year-old mainstream pupil) were not registered. This participant was removed from the data. In total 13.3% of the data were removed. Next, some pupils had a proportion correct of less than .60 on the perceptually easy /b/-/s/ contrast. As this was taken as an indication that those children either did not understand the task or were not concentrating, all data of those children were removed as well (9 children; eight 4-5-year-olds, one 11-year-old; five boys, four girls, two mainstream pupils, five early-English pupils, two bilingual children). For the remaining participants, average proportions correct (and *SD*s) are shown in Table 1.

Information on age, and mean scores on all background measures are shown in Table 2. For the bilingual children, the table also contains information on how often (in % of time) bilingual children were spoken to in English at home (as opposed to Dutch), calculated as the percentage of time English was used by parents during the hours parents and children were together. To calculate this percentage, we first calculated the number of hours that children and each of their parents self-reportedly spent together during the week. Parents indicated what percentage of time they spoke English to their children (as opposed to Dutch or another language). We used this percentage to calculate the number of hours per week that parents spoke English to their children This number was then used to calculate the percentage of time that children were exposed to English by their parents. Next, we also calculated how often (again in % of time) this English came from a native speaker parent (as opposed to a non-native speaker). This was done by 1) checking whether both parents were native speakers, 2) if not, how often the non-native speaker parent used English, and 3) calculating the percentage of time the native speaking parent spent with the children (as opposed to the non-native speaking parent).

**Table 2:** Age and scores on all measures, for each type of English acquisition separately.

		4-5-year-olds			8-9-year-olds			11-12-year-olds		
		Mainstream	Early-English	Bilingual	Mainstream	Early-English	Bilingual	Mainstream	Early-English	Bilingual
English vocabulary (raw score, max. 228)	*N*	15	21	12	15	19	16	18	24	17
	*M*	20.87	26.57	84.83	76.47	72.00	135.19	114.33	108.71	155.59
	*(SD)*	(14.64)	(13.04)	(18.38)	(17.76)	(21.51)	(16.79)	(27.33)	(27.54)	(14.41)
Dutch vocabulary (raw score, max. 204)	*N*	15	21	12	15	19	17	18	24	16
	*M*	69.67	73.52	73.00	113.33	110.16	114.00	132.56	134.79	142.81
	*(SD)*	(10.63)	(13.56)	(11.72)	(6.70)	(6.47)	(11.10)	(12.20)	(9.84)	(11.92)
Intelligence (raw score, max. 41)	*N*	15	21	11	15	19	16	18	24	15
	*M*	11.87	11.62	11.36	20.47	19.11	19.56	21.72	22.83	23.33
	*(SD)*	(4.12)	(2.82)	(3.56)	(3.62)	(3.23)	(3.93)	(5.10)	(4.19)	(5.09)
Age (years)	*N*	15	17	12	15	19	18	18	24	18
	*M*	4.6	4.9	5.1	9.4	9.2	8.5	12.3	12.1	11.1
	*(SD)*	(0.5)	(0.3)	(0.4)	(1.0)	(0.5)	(1.0)	(0.5)	(0.5)	(.8)
Home exposure to English media (hours per week)	*N*	3	15	10	4	11	17	4	6	16
	*M*	4.00	7.93	12.77	5.63	11.82	11.56	29.75	25.46	16.03
	*(SD)*	(1.00)	(5.75)	(6.33)	(3.47)	(7.97)	(10.64)	(41.62)	(11.33)	(8.99)
% exposure to English at home (from parents, either native or non-native speaker of English)	*N*	-	-	10	-	-	18	-	-	16
	*M*	-	-	58.8	-	-	51.4	-	-	53.1
	*(SD)*			(24.3)			(14.8)			(19.1)
% exposure to English from native speaker parent(s)	*N*	-	-	10	-	-	18	-	-	15
	*M*	-	-	84.2	-	-	80.6	-	-	77.3
	*(SD)*			(21.9)			(23.7)			(23.8)

To assess whether the groups were comparable on all measures besides the variables of interest (i.e., the phonetic contrasts), ANOVAs were done with Age (4-5-, 8-9-, and 11-12-year-olds) and Type of English acquisition (mainstream, early-English, bilingual) as independent variables. We used partial eta-squared as a measure for effect size, and followed the general rule of thumb that effects between .02 and .13 are small, those between .14 and .26 are medium, and values of .26 and higher are large [54].

For the dependent variable Age in Months, there was a significant interaction effect between Age (indicated by age groups) and Type of English acquisition (*F*(4, 146) = 5.28, *p* = .001, $\eta_{p}^{2}$= .126, *R*^2^_model_ = .952), Tukey post hoc analyses showed that 8-9-year-old bilinguals were on average younger than mainstream and early-English pupils in the same age group, and 11-12-year-old bilinguals were younger than mainstream pupils in the same age group. For English vocabulary, there was a main effect of Type of English acquisition (*F*(2,148) = 118.56, *p* < .001, $\eta_{p}^{2}$= .616, *R*^2^_model_ = .739). Tukey post hoc tests showed that, as to be expected, bilinguals obtained higher scores than mainstream and early-English pupils. Mainstream and early-English pupils did not differ from each other. As expected, all groups were comparable on Dutch vocabulary (*R*^2^_model_ = .861), and intelligence (*R*^2^_model_ = .561; all *p*s > .05).

The groups were also compared on out-of-school exposure to English. Only a limited number of parents completed the questionnaire. Therefore, Age was not included in the analyses, and an ANOVA with Type of English acquisition as the only independent variable was performed. As expected, the weekly number of hours of out-of-school exposure to English did not differ among the groups (*F*(2,83) = 0.07, *p* > .05, $\eta_{p}^{2}$= .002, *R*^2^_model_ = -.022).

### Perception of phonetic contrasts

**Fig 2:**
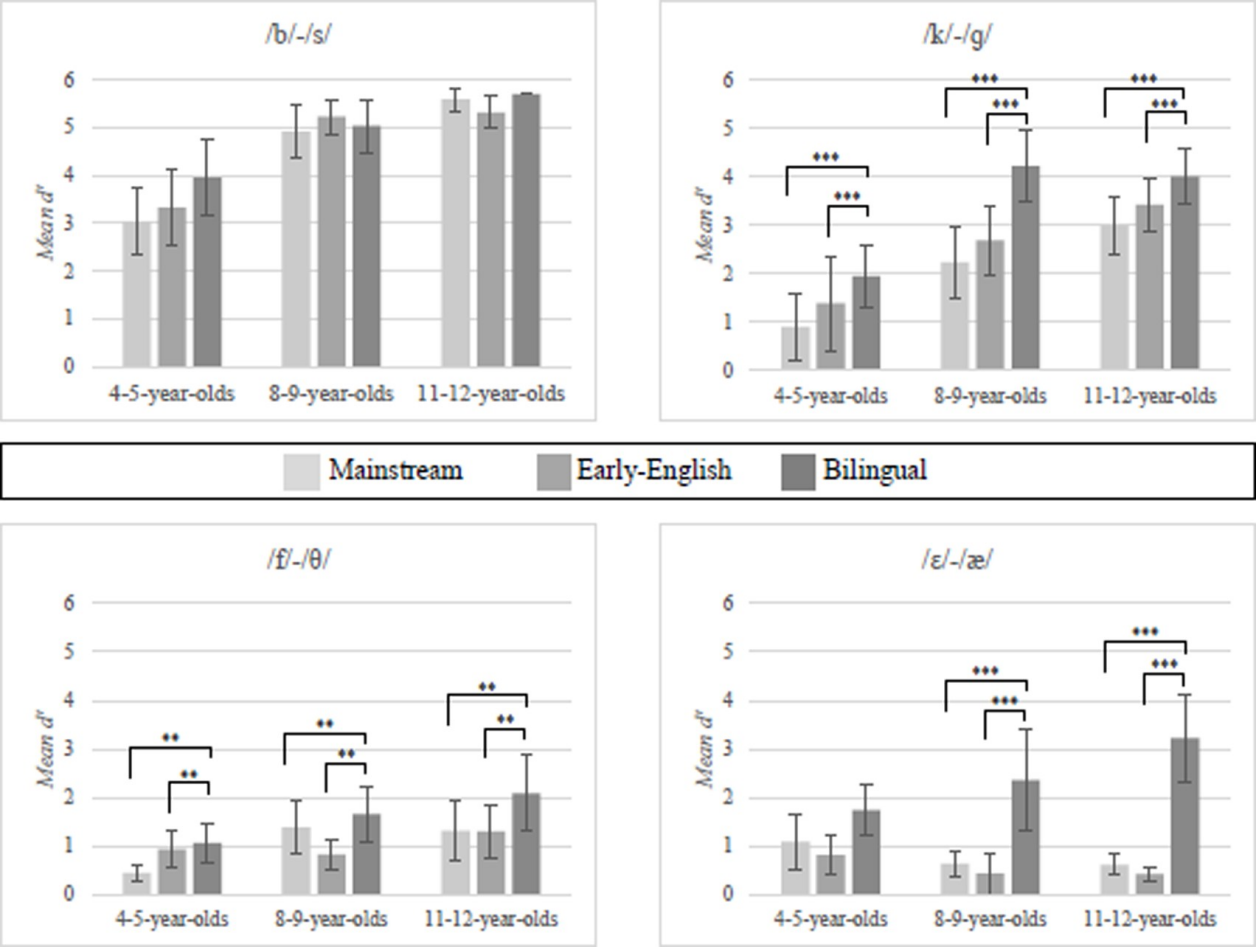
Mean values (with *SD*) for *d’*, for each group separately. Pairwise significant differences (Tukey post hoc tests) are indicated with *p < .05, **p < .01, ***p ≤ .001.

The sensitivity measure *d’* with a correction for near-perfection [55] was calculated for each contrast and for each participant separately, using the dprime.abx function from the psyphy package in platform R (version 3.6.2). One member of each contrast pair was taken as the target (/b/, /k/, /f/, and /ɛ/, respectively). In case the child heard this sound and pressed the correct button, that was counted as a ‘hit’, if the incorrect button was pressed, that was counted as a ‘miss’. If the child heard the other sound (/s/, /ɡ/, /θ/, or /æ/, respectively) and she pressed the correct button, this was counted as a ‘correct rejection, if the wrong button was pressed, it was counted as a ‘false alarm’. The *d’* was then calculated as the difference between z-transformed hits and false alarms. A *d’* of 0 indicates that performance is at chance level, and a higher *d’* value indicates a better ability to discriminate between the two members of the phonetic contrast. Mean values for *d*’ are presented in Fig 2.

An ANOVA with Contrast, Age and Type of English acquisition as fixed factors, and *d’* scores as dependent variable was performed. There were significant main effects for all fixed factors (Contrast: *F*(4,604) = 317.64, $\eta_{p}^{2}$ = .612; Type of English acquisition: *F*(2,604) = 54.78, $\eta_{p}^{2}$ = .154; Age: *F*(2,604) = 72.59, $\eta_{p}^{2}$ = .194; all *p*s < .001, *R*^2^_model_ = .691), and significant interactions between Age and Contrast (*F*(6,604) = 12.09, *p* < .001, $\eta_{p}^{2}$ = .107), and Type of English acquisition and Contrast (*F*(4,604) = 6.32, *p* < .001, $\eta_{p}^{2}$ = .059). The three-way interaction was also significant (*F*(12,604) = 1.97, *p* = .025, $\eta_{p}^{2}$ = .038).

To follow up on this three-way interaction, we performed the same analysis but without Age for each of the age groups separately (see Table 3). The results of all three analyses showed main effects of Type of English acquisition and Contrast, and for 8-9- and 11-12-year-olds a significant interaction effect. Post hoc tests (Tukey HSD) showed that in all three age groups, all English-acquisition-type groups obtained the highest *d’* scores on the /b/-/s/ contrast, followed by the /k/-/ɡ/ contrast, which in turn had higher scores than the other two contrasts. In both the 4-5- and 8-9-year-olds, scores on the /f/-/θ/ and /ɛ/-/æ/ contrasts did not significantly differ. Significant differences among the English-acquisition-type groups emerged for the 11-12-year-olds for the comparison between the /f/-/θ/ and /ɛ/-/æ/ contrasts: For the 11-12-year-old mainstream pupils, scores on the /f/-/θ/ and /ɛ/-/æ/ contrasts did not significantly differ. For the 11-12-year-old early-English pupils, scores on /f/-/θ/ were significantly higher than for /ɛ/-/æ. For the 11-12-year-old bilinguals, to the contrary, scores on /ɛ/-/æ/ were significantly higher than for /f/-/θ/.

**Table 3:** ANOVA on d' scores by contrast (Within-subject) and type of English acquisition (Between-subject).

		4-5-year-olds				8-9-year-olds				11-12-year-olds			
		*df*	*F*	$\eta_{p}^{2}$	Post hoc	*df*	*F*	$\eta_{p}^{2}$	Post hoc	*df*	*F*	$\eta_{p}^{2}$	Post hoc
Intercept		1	189.68***	.513		1	717.54***	.785		1	1250.49***	.846	
Contrast		3	48.28***	.446	b>k>f,e	3	123.34***	.654		3	195.05***	.720	
Type of English acquisition		2	8.77***	.089	B> EE, M	2	17.18***	.149	B> EE, M	2	38.19***	.251	B> EE, M
Contrast x Category		6	0.90			6	3.89**	.106	b>k>f, e (all groups)	6	6.94***	.154	b>k>f,e (M)
													b>k>f>e (EE)
													b>k>f<e (B)
		180				196				228			

N.B.$\eta_{p}^{2}$: < .02-.13 small, .14-.26 medium, >.26 large; M = Mainstream, EE = Early-English, B = Bilingual.

To investigate whether there were effects of bilingualism or Age on the *d’* of the different contrasts, four separate ANOVAs were conducted, one for each contrast. In each of these analyses, Type of English acquisition and Age were fixed factors, and the *d*’ on each of the contrasts was the dependent variable. The results are shown in Table 4.

**Table 4:** ANOVAs with type of English acquisition and age as fixed factors and d’ for each contrast as dependent variable.

		/b/-/s/			/k/-/ɡ/			/f/-/θ/			/ɛ/-/æ/		
	*df*	*F*	$\eta_{p}^{2}$	Post hoc	*F*	$\eta_{p}^{2}$	Post hoc	*F*	$\eta_{p}^{2}$	Post hoc	*F*	$\eta_{p}^{2}$	Post hoc
Type of English acquisition	2	1.32	.017		22.26***	.228	M,EE<B	6.72**	.082	M,EE<B	44.78***	.377	
Age	2	59.32***	.440	4–5<8–9,11–12	48.95***	.393	4–5<8–9<11–12	5.55**	.068	4–5< 11–12	.40	.005	
Age x Category	4	1.28	.033		0.80	.021		1.35	.034		4.13**	.099	4–5: no sign. diff.
													8–9: M, EE<B
													11–12: M,EE<B
Error	151												
*R*^2^_model_		.456			.491			.165			.419		

*p < .05;

**p < .01;

***p < .001

N.B.$\eta_{p}^{2}$: < .02-.13 small, .14-.26 medium, >.26 large; M = Mainstream, EE = Early-English, B = Bilingual.

There was a main effect of Type of English acquisition for all contrasts except /b/-/s/. Bilinguals outperformed both mainstream and early-English pupils on all contrasts except the easy control contrast /b/-/s/, although for the /ɛ/-/æ/ contrast there was a significant interaction with Age; the post hoc tests revealed that only older (i.e., 8-9- and 11-12-year-old) bilinguals significantly outperformed both other groups, whereas the bilinguals in the 4–5 year old group outperformed only the early-English and not the mainstream pupils. Early-English pupils never scored significantly different from mainstream pupils.

Further, there was a significant main effect of Age, showing that younger pupils performed significantly less well than older pupils, for all contrasts except /ɛ/-/æ/. On the /k/-/ɡ/ contrast, all three Ages significantly differed from each other. On the /b/-/s/ contrast the 8-9- and 11-12-year-olds significantly outperformed the 4-5-year-olds, but the former two groups did not significantly differ from each other. On the /f/-/θ/ contrast the 11-12-year-olds outperformed the 4-5-year-olds, but the 8-9-year-olds did not differ from the other two age groups. The same analyses without the children who were exposed to three or four languages revealed the same pattern of results, for all contrasts. When the analyses for *d’* were performed again with the children who had <60% correct on the /b/-/s/ contrast, the results largely remained the same, with only some minor shifts in the outcomes regarding the age differences. We also carried out multi-level analyses to address the nested structure with children under schools. These analyses, with School as a random factor, largely reflected those of the ANOVA, with only minor details in the /k/-/ɡ contrast. We therefore reported the analyses with the simplest structure (the ANOVA).

## Discussion

The aim of this study was to investigate whether Dutch children receiving early-English education were better able to discriminate between English phonetic contrasts than children attending mainstream Dutch primary schools, and whether they were as good as children growing up bilingually in Dutch and English. This question was investigated in three different age groups: 4-5-year-olds who had just started primary school, children who were in the middle of primary school (8-9-year-olds), and 11-12-year-old children who were at the end of primary school.

The first research question was whether the three English-acquisition-type groups of children (mainstream, early-English, bilingual) differed in their abilities to perceive English speech sounds. The hypothesis was that bilingual children might outperform both early-English and mainstream pupils. For the latter two groups, we hypothesized that if anything, the early-English pupils might perform better on the speech perception task than the mainstream pupils. The first part of this hypothesis was confirmed, the second was not. That is, the bilinguals showed greater sensitivity than the two other groups on all the contrast pairs except the easy control contrast /b/-/s/ (i.e., /k/-/ɡ/, /f/-/θ/, /ɛ/-/æ/) but the early-English pupils did not show better performance than the mainstream pupils. The second hypothesis was that there would be age differences in performance on the different contrasts. In line with previous research [14], we found that children between 4 and 12 years old show an increase in phoneme categorization performance. This effect was larger for the /b/-/s/ than for the /k/-/ɡ/ contrast, and smallest for the /f/-/θ/, as shown by a large, medium, and small effect size respectively.

The youngest group (4-5-year-olds) always showed less sensitivity to the contrasts than the older pupils, even to the easy /b/-/s/ contrast. This may be explained by developmental differences in phonemic categorization, as previous research has shown that children continue to refine their phonemic categorization, and that even at the age of 12 they are not as good as adults [14].

The 11- to 12-year-old children did not perform better than 8-to-9-year-old children on the /f/-/θ/ contrast, whereas they did for the /k/-/ɡ/ contrast. PAM predictions for category goodness contrast pairs are that L2 learners learn to hear the differences between the two phonemes after some experience with the L2 [40]. It may be that the relatively small amount of time that mainstream and early-English pupils are exposed to English is enough for an initial growth in sensitivity towards the /f/-/θ/ contrast, but not enough for a further improvement. On the other hand, the oldest group of bilingual children did not perform significantly better than the 8-9-year-old group either. Even adult English native speakers find /f/ and /θ/ to be perceptually confusable [41,47]. Therefore, the difficulties that the early-English pupils experienced with this contrast are hardly surprising. In fact, it could be that between 8–9 years and 11–12 year, perception of this contrast does not measurably develop.

For the /ɛ/-/æ/ contrast, mainstream and early-English pupils of all ages performed at chance level and showed no improvement at all with age. According to the predictions outlined in PAM-L2 [40], both members of such a single category contrast should be perceived as a similarly good or poor exemplar of the closest native phoneme and therefore learners are not likely to learn to distinguish between the two members [40]. We proposed that /ɛ/ and /æ/ should be perceived as an exemplar of the Dutch /ɛ/, resulting in pupils of all ages performing at chance level. Our results seem to confirm this reasoning.

Our third hypothesis was that the early-English and mainstream pupils’ perceptual difficulty would vary across the phonetic contrasts tested and depend on the degree of similarity of those phonemes in English and Dutch. This hypothesis was based on Best and Tyler [40] who predict that L2 learners will have difficulty perceiving non-native speech contrasts, especially when one or both members are very similar but not identical to a native phonetic category. Since the bilingual children were native speakers of both Dutch and English, we predicted that their perception should generally be more accurate. We focused on four contrasts, which we expected to vary in difficulty for the early-English and mainstream pupils: /b/-/s/ (easy), /k/-/ɡ/ (intermediate), /f/-/θ/ (hard), and /ɛ/-/æ/ (very hard). Our hypothesis that perceptual difficulty would vary across the phonetic contrasts was confirmed. All children (mainstream, early-English, and bilingual) performed best on the /b/-/s/ contrast. All Type-of-English-acquisition groups performed relatively well on the /k/-/ɡ/ contrast too, although scores were significantly lower than on the /b/-/s/ contrast. We expected the k/-/ɡ/ contrast to be of intermediate difficulty, as Dutch, first, contrasts between voiceless-voiced plosives at other places of articulation and second, contains /ɡ/ in loan words and third, contains it as an allophone of /k/. Confirming our hypothesis that L2 acquiring pupils would have less difficulty with the /k/-/ɡ/ contrast than with the /f/-/θ/ or ɛ/-/æ/ contrast, mainstream and early-English pupils performed worst on these two latter contrasts. For the oldest group of bilingual children, the performance pattern was different: they performed worse on /ɛ/-/æ/ than on /k/-/ɡ/, but they had even more difficulty with /f/-/θ/.

PAM-L2 does not differentiate between novel L2 sounds that listeners are entirely unfamiliar with, and sounds that they do have some L1 experience with, because they represent a system gap such that they are familiar with relevant features, or because they occur in the L1 in loan words or as allophones. In this study, we tested perception of two contrasts of the category goodness type, /k/-/ɡ/ and /f/-/θ/. Our results, with better performance for /k/-/ɡ/ than for /f/-/θ/, imply that L2 learners have less difficulty distinguishing between two members of a contrast (here /k/-/ɡ/) when they have L1 experience with the type of contrast (in this case concerning stop voicing) or with the ‘absent’ phoneme itself (because it occurs in L1 as a marginal phoneme and an allophone), than between those of a category goodness contrast that does not have this benefit of L1 familiarity (here /f/-/θ/). An alternative explanation for the difference between pupils’ performance on the /k/-/ɡ/ contrast and the /f/-/θ/ contrast could be that the difference between /f/-/θ/ is intrinsically difficult, in line with the bilinguals’ relatively low performance on this contrast. A study with English monolingual infants investigated the hypothesis that it is easier to discriminate between between-organ articulation contrasts (in this study /f/-/θ/) than within-organ articulation contrasts (in this study /s/-/θ/), but this hypothesis was not confirmed [58]. According to this hypothesis, /f/-/θ/ should have been easier to distinguish than /k/-/ɡ/. That was, however, not the case. It was even the other way around. We therefore reject this alternative explanation, and adhere to our explanation that even though both the /k/-/ɡ/ and the /f/-/θ/ contrast are Category Goodness contrasts, they are fundamentally different. Further research on adult and child L2 learners’ performance on different types of category goodness contrasts is needed to confirm whether contrasts such as the /k/-/ɡ/ in this study are treated differently in L2 learning.

The overall aim of this study was to investigate if children who are learning English by means of an early-English educational programme outperform their peers who are not enrolled in such a programme. Early-English pupils did not outperform mainstream pupils on any of the non-native contrasts. Note that problems with performance on these contrasts cannot be due to the difficulty level of the task itself: Even children in the youngest group were capable of performing the task, as indicated by their performance on the easy /b/-/s/ contrast. There are several explanations possible for these outcomes. First of all, it may be that the limited exposure to English at school is simply not enough for early-English pupils to learn the difference between English speech sounds, or for pupils’ knowledge of English in general to develop.

Second, it could be that the exposure to English that children get at home is more important than the input at school. The available parental questionnaire data show that both mainstream and early-English pupils had considerable exposure to English media at home, and that early-English pupils had more exposure to English out of school than in school. Unfortunately, information about out-of-school exposure was not available for all children. Exploratory partial correlation analyses between out-of-school exposure and performance on each of the contrast pairs while controlling for age revealed no significant relations between the two variables (for /b/-/s/: *r* = 0.183; /k/-/ɡ/: *r* = .048; /f/-/θ/: *r* = .005; /ε/-/æ/: *r* = -.007; all *p*s > .05). Although for a correlation analysis to be more informative, these results suggest that out-of-school exposure to English does not contribute to pupils’ perception of English contrasts.

An alternative explanation for the lack of a difference between the early-English and mainstream pupils’ results could be that type of exposure that pupils receive at school does not contribute to the perception of L2 speech contrasts. As outlined in the introduction, English education in Dutch schools is generally aimed at improving pupils’ oral proficiency, and specifically at improving their speaking and listening skills and their vocabulary knowledge [35]. Its goal is furthermore to advance pupils’ understanding of spoken English and written English texts, and for them to become able (and confident) to communicate in English [34]. As being able to hear and produce the differences between English speech sounds is not one of the goals for English education, explicit instruction on perception or pronunciation of English phonemes appears not to be part of the educational curriculum. In addition, English lessons are generally provided by the regular classroom teacher [27]. Although the required English proficiency level is B2 (intermediate), teachers’ proficiency levels vary largely, with a substantial number of teachers not obtaining the required level [27]. As a consequence, teachers may not correctly produce (some of the) English speech sounds. We did not measure the teachers’ ability to produce the speech contrasts in question, so we do not know whether pupils received the correct input. Given that Dutch speakers of English have difficulty with pronouncing /θ/ [56], /ɛ/, and /æ/, and, albeit to a lesser extent, /ɡ/ [42], it may thus well be that teachers were not able to produce these sounds. They may thereby providing children with Dutch-accented English, in which these sounds were not pronounced correctly, and limiting the possibilities for pupils to implicitly learn to distinguish between those speech sounds. However, even if some teachers were able to produce the speech sounds correctly the question is to what extent that will positively influence pupils’ speech perception skills: pupils usually have two class teachers, have different teachers every school year, and receive additional English input via English media, and the influence of an individual teacher is probably limited.

The results of this study are not in line with the premise that early-English education has a positive effect on pupils’ English language knowledge. We did not find any advantages for the early-English group compared to the mainstream group in their perception of English speech sounds. We also measured pupils’ English vocabulary, to examine whether early-English pupils’ English vocabulary is greater than that of mainstream pupils. Contrary to what might be expected, we did not find advantages for the early-English group in this domain either. As we tested four different speech contrasts and receptive vocabulary only, we cannot rule out the possibility that early-English education may be beneficial for other domains of English language development, such as active vocabulary or grammar knowledge. Nevertheless, these outcomes are important for policy makers, who base the implementation of English educational programmes premises that early-English education will almost automatically benefit pupils’ English language development. Such premises are often based on findings that show that children who are growing up bilingually in naturalistic settings successfully master two languages [49]. There are numerous ways (e.g. learning two languages at home), however, in which growing up with two languages differs from learning a new language at school while one language has already largely developed [49]. This study shows that early-English education, as it is currently implemented in the Dutch school system, may have very limited effects on pupils’ abilities to perceive differences between English speech contrasts–at least on the ones we tested in this study. This finding does not rule out that Early-English education might be beneficial to pupil’s perception of English speech contrasts if teaching perception and pronunciation of non-native phonemes receives a more prominent role in the curriculum. Research with adults has shown that for those learning an L2 after their L1 has already been largely established, both perception and pronunciation teaching may benefit the acquisition of non-native speech contrasts [57,58], even when the teacher is not a native speaker herself [59].

As our study was not longitudinal, we cannot rule out the possibility that early-English pupils older than four but younger than eight (i.e., between the ages at which we tested them) have an advantage in vocabulary or in the perception of the /k/-/ɡ/ or /f/-/θ/ contrast that the mainstream pupils later catch up on. This cross-sectional study nevertheless provides a first and important exploration of the question whether early-English programmes are beneficial for pupils’ perception of English speech contrasts. Because we assessed pupils who were at the end of primary school, we could show that even after eight years of English lessons, pupils are not better able to perceive English speech contrasts that do not exist in Dutch than pupils who started English education little more than a year before being tested. By investigating multiple contrasts that varied in difficulty, we have shown that young learners of English, both in early-English and in mainstream education, did manage to learn to distinguish the difference between members of some non-native phoneme pairs, but only those that were easy or of moderate difficulty.

## Conclusions

This study shows that early-English education is not beneficial to non-native speech perception: Dutch children who get a maximum of two hours of English lessons per week from the moment they enter primary school at the age of four are not better able to perceive the difference between members of English contrasts than children who start in the penultimate grade of primary school. Children growing up bilingually with Dutch and English were, however, better at hearing the differences in all phonetic contrasts except the easy control contrast than the children who did not grow up bilingually at home. Starting to acquire English in an instructed setting at a young age thus appears not to be beneficial for learning to perceptually distinguish non-native contrasts. For pupils to learn to perceive non-native speech contrasts, perception (and pronunciation) instruction should get a more prominent role in the early-English curriculum.

## Supporting information

S1 Data How bilingual are early-English learners. (SAV)
